# Retraction: Antidepressant-Like Activity of YL-0919: A Novel Combined Selective Serotonin Reuptake Inhibitor and 5-HT_1A_ Receptor Agonist

**DOI:** 10.1371/journal.pone.0304643

**Published:** 2024-05-24

**Authors:** Hong-xia Chen, Zeng-liang Jin, Li-ming Zhang, Rui Xue, Xiao-dan Xu, Nan Zhao, Zhi-kun Qiu, Xian-wang Wang, You-zhi Zhang, Ri-fang Yang, Yun-feng Li

After this article [1] was published, the corresponding author requested its retraction stating that the authors no longer have confidence in the article’s results. They have since determined that results reported in Tables 3 and 4 are unreliable due to methodological errors, and that YL-0919 is not a selective SERT/5-HT_1A_ receptor agonist as reported in the article.

Specifically, the collected membrane protein used to evaluate the affinity of the compound YL-0919 for 5-HT transporter (SERT) and 5-HT_1A_ receptor was extracted from rat brain tissue over a period of 7–9 hours and the membrane proteins are not expected to maintain their naive biological activity under the experimental conditions used. In experiments conducted after the publication of [1], the homogeneous membrane protein showed time-dependent degradation, leading to a continuous decrease in total binding per loading sample. The negative control also showed a high affinity binding curve characteristic resulting in false positive results in the SERT and 5-HT_1A_ binding assay.

An expert member of the Editorial Board confirmed that the experimental conditions described in [1] are likely to result in significant degradation. They further noted that the buffer used in the experiments affects high affinity binding by known ligands and therefore the results obtained using the buffer should be considered inconsistent and not reliable.

In light of the above issues, the authors retract this article. The authors and the publisher apologize that these issues were not identified prior to the article’s publication.
